# Constructing a Segregated Magnetic Graphene Network in Rubber Composites for Integrating Electromagnetic Interference Shielding Stability and Multi-Sensing Performance

**DOI:** 10.3390/polym13193277

**Published:** 2021-09-26

**Authors:** Jian Wang, Baohua Liu, Yu Cheng, Zhenwan Ma, Yanhu Zhan, Hesheng Xia

**Affiliations:** 1 College of Food and Biological Engineering, Chengdu University, Chengdu 610106, China; liubaohua@cdu.edu.cn; 2 State Key Laboratory of Polymer Materials Engineering, Polymer Research Institute, Sichuan University, Chengdu 610065, China; yuchengnero@163.com (Y.C.); mazhenwan1289@163.com (Z.M.); 3 School of Materials Science and Engineering, Liaocheng University, Liaocheng 252059, China

**Keywords:** reduced graphene oxide, natural rubber, electromagnetic interference shielding stability, multi-sensing property, segregated network

## Abstract

A flexible, wearable electronic device composed of magnetic iron oxide (Fe_3_O_4_)/reduced graphene oxide/natural rubber (MGNR) composites with a segregated network was prepared by electrostatic self-assembly, latex mixing, and in situ reduction. The segregated network offers the composites higher electrical conductivity and more reliable sensing properties. Moreover, the addi-tion of Fe_3_O_4_ provides the composites with better electromagnetic interference shielding effectiveness (EMI SE). The EMI shielding property of MGNR composites is more stable under tensile deformation and long-term cycling conditions and has a higher sensitivity to stretch strain compared with the same structure made from reduced graphene oxide/natural rubber (GNR) composites. The EMI SE value of MGNR composites reduces by no more than 2.9% under different tensile permanent deformation, cyclic stretching, and cyclic bending conditions, while that of GNR composites reduces by approximately 16% in the worst case. Additionally, the MGNR composites have a better sensing performance and can maintain stable signals, even in the case of cyclic stretching with a very small strain (0.05%). Furthermore, they can steadily monitor the changes in resistance signals in various human motions such as finger bending, wrist bending, speaking, smiling, and blinking, indicating that the MGNR composites can be used in future wearable electronic flexibility devices.

## 1. Introduction

Rubber-based composite materials, especially graphene rubber composite materials, have excellent flexible, thermoelectric, sensing, and morphologically controllable properties. These materials are ideal for smart, flexible, wearable electronic devices that have been studied by many researchers [1,2,3,4,5]. However, a certain amount of electromagnetic interference (EMI) occurs between electronic products, which will affect the performance of this kind of device. It is therefore of great interest to prepare flexible electronic materials with an excellent EMI shielding performance [6,7,8].

Enhancing the electrical conductivity of the material, adding magnetic particles, and increasing the thickness of the material are effective methods for improving the EMI shielding properties of rubber materials [9,10,11,12,13,14,15,16]. For rubber matrix materials, the preparation of composite materials with a segregated or 3D network structure can increase the conductivity of the rubber material by several orders of magnitude, thereby improving the EMI shielding performance of the composites [17,18,19,20,21]. Jia et al. [18] prepared carbon nanotube/natural rubber composites with a flexible network structure. The prepared composites have an excellent EMI shielding performance and can effectively maintain this performance under different stretching, bending, and twisting conditions. Adding magnetic particles, such as Fe_3_O_4_, is also an effective way to improve the EMI shielding of the composites [19,22,23,24,25]. Zhu et al. [19] prepared an innovative Fe_3_O_4_/graphene foam/poly dimethylsiloxane (PDMS) material that is assembled by anchoring the magnetic Fe_3_O_4_ particles onto the highly electronically conductive 3D graphene foam. Due to the synergistic effect between Fe_3_O_4_ nanoparticles and graphene foam, the EMI SE of the composite (~1.0 mm) increases from ~26.6 dB for graphene foam/PDMS composite to ~32.4 dB in the frequency range of 8.2–12.4 GHz. Furthermore, after repeatedly bending for 10,000 cycles, the EMI SE of the Fe_3_O_4_/graphene foam/PDMS composite still reaches up to 29.4 dB.

Nowadays, flexible, wearable electronic devices with superior sensing properties that can monitor the human body’s physical health, walking movement, state changes, etc., play a significant role in the field of smart products in our daily lives [26,27,28,29,30,31,32,33,34,35,36,37]. Additionally, it is important to prepare flexible composites with both excellent EMI shielding and sensing properties. This will lead to better functionality for flexible, wearable electronic materials. Wang et al [6]. prepared flexible and conductive graphene oxide (GO)/cellulose nanofibril (CNF)/nitrile rubber (NBR) composite films with a 3D network conductive structure. The flexible composite film can be used as a piezoresistive sensor for wearable devices, which can hold precise current signals and respond to finger motions. The specific 3D network structure also enhances the EMI shielding effectiveness of the composites up to 25.81 dB in the X band, which makes them better for use in wearable and portable medical equipment and electronic devices.

For the wearable electronic device composites, it is also very important to make the composites with excellent EMI shielding stability and high strain sensing properties even with very low strain. In our previous work, we prepared rGO/Fe_3_O_4_/NR composites (MGNR) with a segregated network that exhibit excellent EMI shielding performance [17]. The existence of Fe_3_O_4_ particles anchored on the segregated graphene network will also bring the composites great potential in reliable resistance change during the strain process, even with a very small strain, as well as excellent EMI shielding stability performance. So based on previous research, we further discuss the stability of the EMI shielding performance of the composites under different tensile deformations and long-term cycling conditions, as well as their sensing properties following cyclic stretch strain and the effect that the addition of Fe_3_O_4_ particles has on human motion detection. The results show that the MGNR composites have excellent sensing properties, even with very low strain, as well as EMI shielding stability after tensile deformations, cyclic stretching, and bending, and can be used in modern smart flexible materials.

## 2. Materials and Methods

### 2.1. Materials

Natural rubber latex (solid content: 60 wt%) was provided by Chengdu Fangzheng Co., Ltd. (Chengdu, China). Graphene oxide (SE2430) was supplied from The Sixth Element Materials Technology Co., Ltd (Changzhou, China). Hydrazine hydrate, ferric chloride hexahydrate (FeCl_3_∙6H_2_O), green vitriol (FeSO_4_∙7H_2_O), ammonium hydroxide and hydrazine hydrate were purchased from Chendu Kelong Chemical Reagent Company (Chengdu, China). All other reagents, including sulfur, zinc oxide, accelerator N-cyclohexyl-2-benzothiazolesulfenamide (CBS), 2-mercaptobenzothiazole (MBT), antioxidant (4010NA), stearic acid, and emulsifier OP, are commercially available.

### 2.2. Synthesis of MGNR and GNR Composites

The synthesis of the MGNR and GNR composites was described in our previous work [17]. Firstly, graphene oxide was dispersed in water (3 mg/mL) for 2 h in an ultrasonic bath to form a stable graphene oxide (GO) dispersion. Afterward, a mixed solution of FeCl_3_·6H_2_O and FeSO_4_·7H_2_O with a molecule ratio of 2:1 was dispersed into the above ultrasound GO solution. The mass ratio of GO: FeCl_3_·6H_2_O:FeSO_4_·7H_2_O was 1:5:2.56. After ultrasonicating for 30 min, an aqueous ammonium hydroxide solution was slowly injected into the mixed solution until the pH of the solution reached 12. After 1 h, Fe_3_O_4_/GO hybrids were obtained and the NR latex was dispersed into the Fe_3_O_4_/GO solution by ultrasonication for 30 min to obtain NR/Fe_3_O_4_/GO latex. Hydrazine hydrate was injected into the NR/Fe_3_O_4_/GO latex and the mixture was in situ reduced with ultrasound at 60 °C for 2 h. The sulfur and other additives formed an aqueous suspension with a concentration of 4 mg·mL^−1^ (the content of NR was fixed at 100 phr, the content of rGO was at 4, 6, 8, or 10 phr, zinc oxide was at 5 phr, sulfur was at 2.8 phr, antioxidant 4010NA was at 3 phr, stearic acid was at 3 phr, accelerator MBT was at 0.1 phr, accelerator CBS was at 1.4 phr, and emulsifier OP was at 2 phr), which was dispersed into the Fe_3_O_4_/rGO/NR latex. Finally, the mixed latex was coagulated. After filtration, the solid mixture was dried in a vacuum oven at 65 °C for 4 h. The composites were compression molded and vulcanized at a temperature of 150 °C and a pressure of 10 MPa for 5 min to obtain the composites. The obtained MGNR composites were designated MGNR-x, in which x is 4, 6, 8, or 10 depending on the rGO content. GNR composites were prepared by the same process.

### 2.3. Characterization

TEM images of MGNR and GNR composites were taken using an FEI Tecnai G2 F20 S-TWIN (FEI Inc., Hillsboro, OR, USA) transmission electron microscope. Electrical conductivity was detected by employing a picometer (Keithley 2400, Keithley Instruments Inc., Solon, OH, USA) system. The electromagnetic interference shielding properties of the composites were evaluated by a vector network analyzer (Agilent N5247A, Agilent Technologies Inc., Santa Clara, CA, USA) in transmission–reflection mode. The scattering parameters in the frequency range of 8.2–12.4 GHz (X-Band) were recorded.

For the tensile permanent deformation test, rectangular GNR-6 and MGNR-6 samples (45 × 15 × 0.6 mm^3^) were fixed to each end of a self-made permanent deformation machine at a distance of 15 mm. The samples set aside 15 mm in the middle to test the tensile permanent deformation of the composites. The GNR and MGNR samples were stretched to a length of 25% (3.75 mm), 50% (7.5 mm), and 75% (11.25 mm) for 12 h. Finally, the samples were removed and left for 12 h, testing the enhanced length and then taking the middle part of the samples to test the electrical conductivity and EMI shielding properties. In order to better explain the changes in the segregated network before and after the tensile permanent deformation test, a GNR-6 sample (45 × 15 × 0.6 mm^3^) was subjected to tensile permanent deformation with 100% strain (15 mm stretched length). Additionally, the morphology of the GNR-6 composites before and after being subjected to tensile permanent deformation were characterized by TEM (FEI Tecnai G2 F20 S-TWIN, Hillsboro, OR, USA).

For stretching fatigue properties, the rectangular GNR-6 and MGNR-6 samples (45 × 15 × 0.6 mm^3^) were cyclically stretched 250, 500, 1000, 1500, and 2000 times on the MTS810 fatigue tester (MTS Corporation, Eden Prairie, MN, USA, 15 mm test length with ±3.75 mm amplitude). The middle parts were then taken to test the electrical conductivity and EMI shielding properties. For bending fatigue properties, the MGNR-6 and GNR-6 samples (45 × 15 × 0.6 mm^3^) underwent cyclic bending at a certain bending frequency (3 Hz) and angle 250, 500, 1000, 1500, and 2000 times. The electrical conductivity (parallel direction) and EMI shielding performance were then measured.

For cyclic tensile sensing measurements, different rectangular GNR and MGNR samples (45 × 10 × 1 mm^3^) were fixed to an MTS CMT-4000 universal (MTS Corporation, Eden Prairie, MN, USA) testing machine (reserve 25 mm in the middle) and the samples were stretched at a tensile speed of 10 mm/min. The electrical resistance of the samples during the tensile process were recorded with the Keithley 6485 picometer (Keithley Instruments Inc., Solon, OH, USA). The gauge factor (*GF*), which can measure the sensing properties of the material, is obtained from Equation (1) [38,39]:

(1) $$GF=\frac{\Delta R/R_{0}}{\varepsilon }$$

where Δ*R*/*R*_0_ is the relative change in the resistance and *ε* denotes the applied stretching strain.

For human sensing measurements, the samples were fixed onto different human body parts (fingers, arms, throat, mouth corners, and eyes) and the electrical resistance change under different human motion conditions (finger bending, wrist bending, talking, smiling and blinking) was measured at least six times.

## 3. Results and Discussion

### 3.1. Morphology of GNR and MGNR Composites

**Figure 1:**
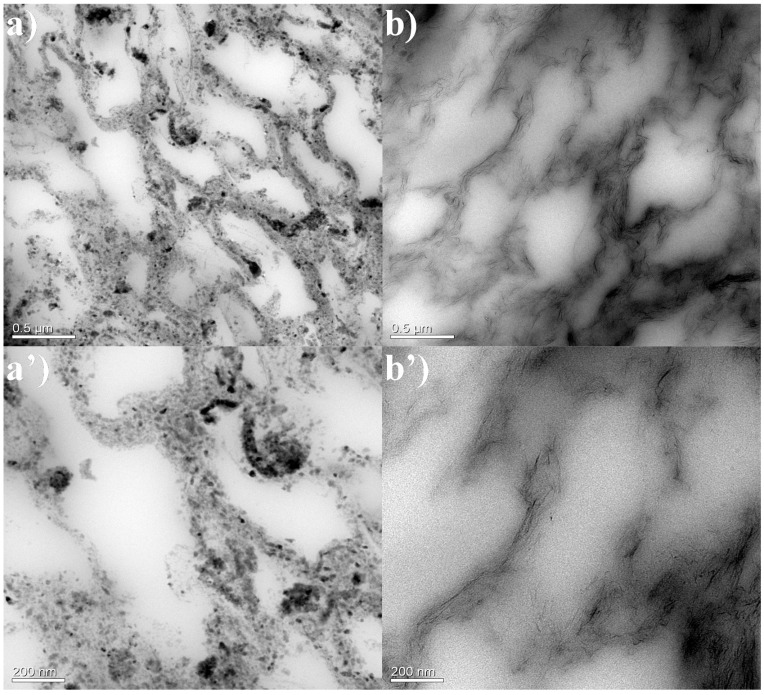
TEM images of MGNR-6 (**a**,**a’**) and GNR-6 composites (**b**,**b’**).

In our previous study, it was proven that the discrete and spherical Fe_3_O_4_ particles are homogeneously anchored on the surface of the flake-like shape rGO sheets, suggesting a strong interaction between the Fe_3_O_4_ nanoparticles and the rGO sheets [17]. Additionally, the morphology of GNR and MGNR composites was characterized by TEM in Figure 1. From Figure 1a,a’, we can clearly see that the rGO flakes with anchored Fe_3_O_4_ particles coated the surface of the rubber matrix and connected to form a segregated conductive network in all MGNR composites. This gives the MGNR composites ferromagnetic properties, which are very important for enhancing the EMI shielding properties of MGNR composites [17]. The GNR and MGNR composites were both made by ultrasonically assisted latex mixing and an in situ reduction process; it is worth noting that the similar segregated structure in the MGNR and GNR composites exist, which can be seen in Figure 1. The segregated structure can greatly enhance the electrical properties of the composites, causing an improvement in the EMI shielding performance. Additionally, the structure of the segregated network can be changed during different tensile or human processes, which can alter the resistance during the above situations [40]. As a result, the composites have reliable sensing properties.

### 3.2. The Stability of EMI Shielding and Electrical Conductivity Properties of MGNR and GNR Composites under Different Mechanical Deformation

In our previous work, we proved that the addition of Fe_3_O_4_ particles decreases the electrical conductivity and increases the EMI shielding properties of the composites [17]. The specific data about the electrical conductivity and EMI shielding properties of different GNR and MGNR composites are shown in the supporting information. Specifically, the EMI SE value of the MGNR-10 composites is 42.6 dB at 8.5 GHz, while that of the GNR-10 composites is only 32.4 dB at the same frequency, which is shown in Figure S2a. This is because the Fe_3_O_4_ particles cause the composites to have more magnetic field interactions with natural resonance, exchange resonance, and eddy currents [41,42]. Additionally, the addition of Fe_3_O_4_ can enhance the interface polarization relaxation between the fillers and the rubber matrix, which increases the transmission path of electromagnetic waves between composite materials, consequently increasing the possibility of the attenuation of incident waves [43,44,45]. In terms of the EMI shielding mechanism, it is the absorption efficiency, not the reflection efficiency, that contributes more to the EMI SE of the MGNR composites, absorbing most of the electromagnetic radiation that is then dissipated in the form of heat [17]. Furthermore, the specific EMI SE (EMI SE divided by sample thickness) of MGNR-10 composites was 21.3 dB∙mm^−1^, which is competitive with the reported EMI shielding performance properties of polymer/rGO or polymer/Fe_3_O_4_/rGO composites [24,42,46,47,48]. 

**Figure 2:**
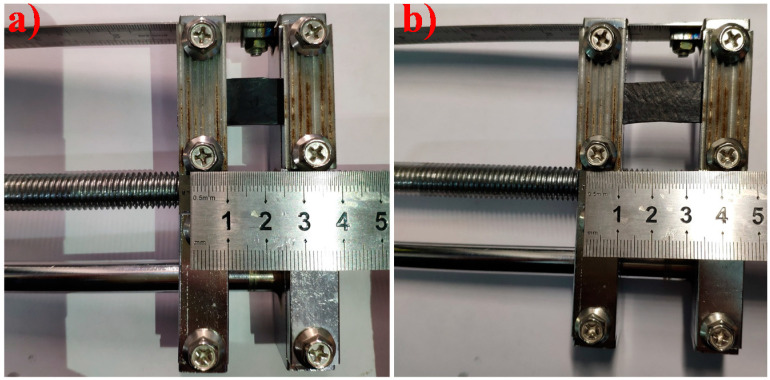
The machine that used to test the rubber permanent deformation properties (**a**) original; and (**b**) pulled for 50% strain.

**Figure 3:**
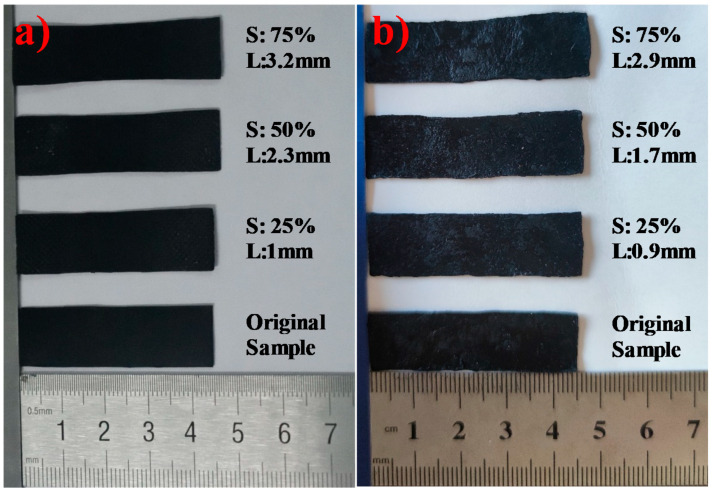
The enhanced length of the GNR-6 (**a**) and MGNR-6 (**b**) composites after treatment by permanent deformation with different strain (S presents the tensile strain elongation and L presents the enhanced length).

Apart from good EMI shielding properties, EMI shielding stability under different cyclic stretching, cyclic bending, and tensile permanent deformation is also important in flexible shielding materials. First, we tested the stability of the EMI SE value under tensile permanent deformation. The rectangular GNR-6 and MGNR-6 samples (45 × 15 × 0.6 mm^3^) were held over a length of 15 mm in the middle of a self-made tensile permanent deformation machine and were stretched by 25% (3.75 mm), 50% (7.5 mm), and 75% (11.25 mm) of the original length. The specific experiment schematic can be seen in Figure 2. The tensile permanent deformation results are shown in Figure 3**,** in which it can be seen that the enhanced length of the GNR composites that have been treated by permanent deformation under strains of 25%, 50%, and 75% are 1 mm, 2.3 mm, and 3.2 mm, respectively. Meanwhile, the enhanced length of MGNR composites under strains of 25%, 50%, and 75% are 0.9 mm, 1.7 mm, and 2.9 mm. The enhanced permanent deformation length of the treated MGNR composites is slightly smaller than that of GNR composites. This may be due to the fact that the addition of Fe_3_O_4_ particles enhances the stiffness of the composites, and therefore reduces the permanent deformation length.

**Figure 4:**
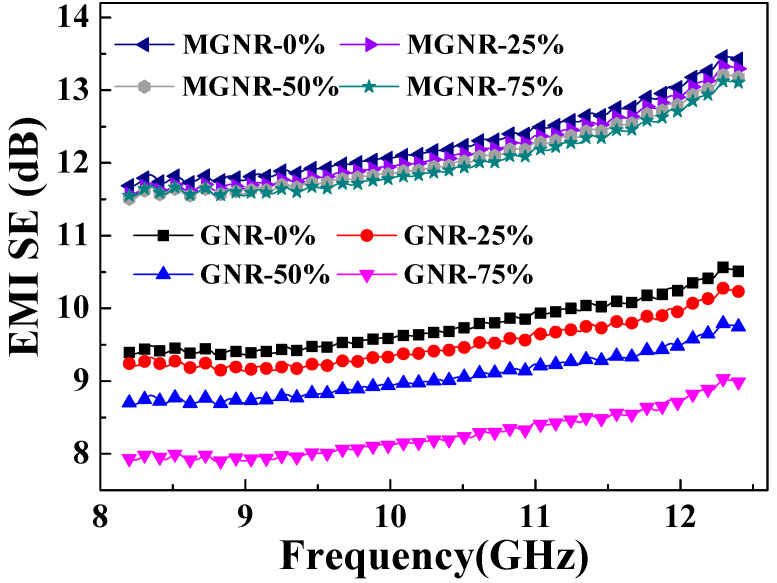
EMI SE of the MGNR-6 and GNR-6 composites which were treated for permanent deformation with different strain (the thickness was 0.6 mm).

**Figure 5:**
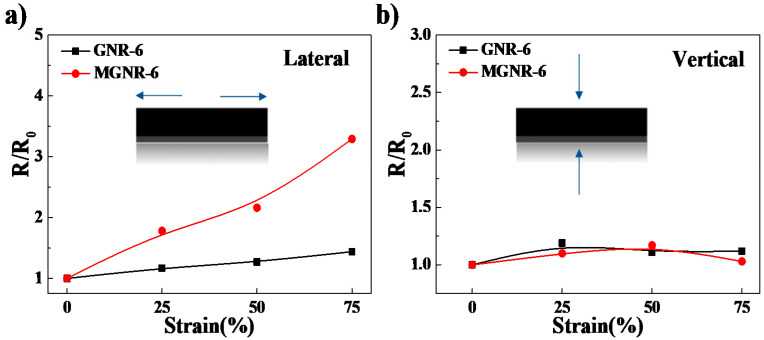
The *R*/*R*_0_ as a function of the permanent deformation with different strain for MGNR-6 and GNR-6 composites: (**a**) lateral direction; (**b**) vertical direction.

The EMI shielding performance and electrical conductivity of different samples that were treated under different tensile permanent deformations are shown in Figure 4 and Figure 5. From Figure 4, we can see that the average EMI SE value of MGNR-6 composites that were treated under different tensile permanent deformations decreased by a small amount (less than 2.2%) at all frequencies, indicating that MGNR has good EMI shielding stability. Meanwhile, the average EMI SE value of the GNR-6 composite decreased markedly, by up to 16% in the worst case. For the change in the electrical conductivity of MGNR and GNR composites, we observed opposite results from the two directions, which can be seen in Figure 5. In the stretching direction, the conductivity of the MGNR composites decreased more (higher *R*/*R*_0_ value) than the GNR composites, while the conductivity performance in the vertical stretching direction barely changed.

**Figure 6:**
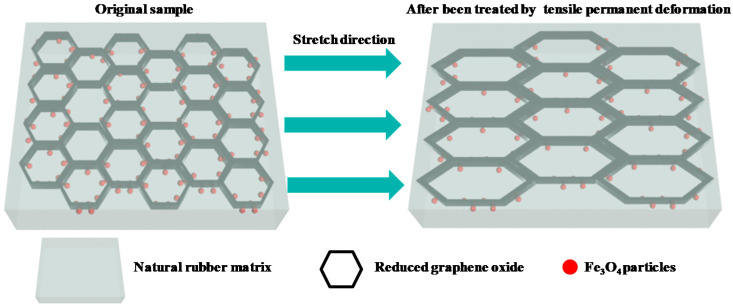
The change of the segregated network along the stretched direction after being treated by tensile permanent deformation of MGNR composites.

**Figure 7:**
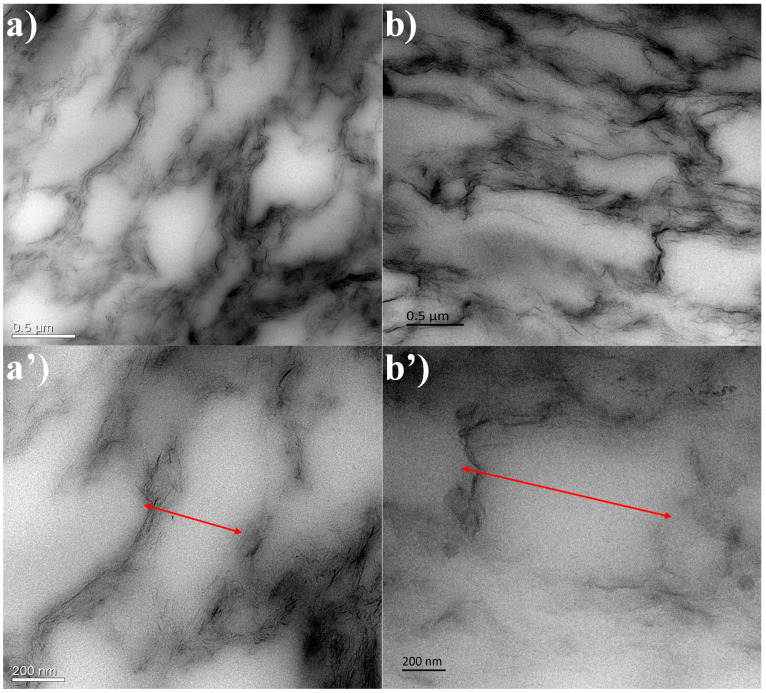
The TEM images of the GNR-6 composites before (**a**,**a’**) and after (**b**,**b’**) the rubber permanent deformation with 100% strain (15 mm stretched length).

In Figure 1, we can see that the segregated network structure has an important influence on the electrical conductivity and EMI shielding performance of the GNR and MGNR composites. The tensile permanent deformation changes the segregated network along the stretched direction and affects the properties of the composites, which is demonstrated in Figure 6. In order to better explain the changes in the segregated network before and after the tensile permanent deformation test, we took the GNR-6 sample as an example to compare the TEM image (Figure 7) of the original GNR-6 sample and the GNR-6 sample that had been subjected to permanent deformation with 100% strain (15 mm stretched length). From Figure 7, we can see that after tensile permanent deformation, the segregated network becomes longer in the stretching direction (the red arrow), which causes electrons to take a longer path to go through the rubber network (or less conductive rGO particles in the same length area). This leads to worse electrical conductivity for the GNR and MGNR composites [49,50]. The electrical conductivity of the GNR composites mainly determines the EMI shielding performance, so the decline in electrical conductivity greatly reduces the EMI shielding properties of GNR composites. However, for the MGNR composites, the outstanding EMI shielding performance is determined by good electrical conductivity and excellent magnetic properties. Although the electrical conductivity is reduced, the addition of Fe_3_O_4_ gives the materials higher magnetic permeability, and thus greater magnetic loss. As a result, the MGNR composites can efficiently absorb electromagnetic wave radiation, maintaining their excellent EMI shielding properties [48].

For the electrical conductivity in the stretching direction for MGNR composites that have been treated by tensile permanent deformation, the lengthening of the segregated network (or the decreased conductive rGO particles in the same length MGNR area) and the addition of non-conductive Fe_3_O_4_ particles will both increase the electronic transmission path in the conductive network and prevent electronic transmission between the conductive rGO nano-platelets. Therefore, the electrical conductivity of the MGNR composites decreases more (lager *R*/*R*_0_ value) than that of the GNR composites after tensile permanent deformation. However, from another point of view, the MGNR composites have higher electrical resistance changes under the same tensile strain deformation, which shows that, compared to the GNR composites, the MGNR composites have better sensing properties in the same strain process. In the vertical direction, the electrical conductivity of both GNR and MGNR composites did not change much, which also proves that the change in the segregated network in the stretching direction is the main driver affecting the electrical conductivity of the composites.

**Figure 8:**
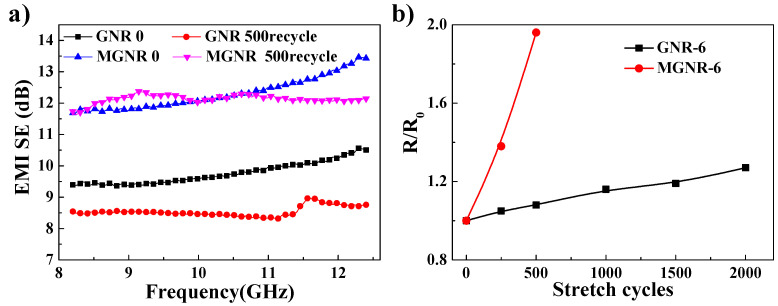
(**a**) The EMI SE of the MGNR-6 and GNR-6 composites before and after stretching for 500 cycles; (**b**) the *R/R*_0_ as a function of the stretching cycles for MGNR-6 and GNR-6 composites (the thickness was 0.6 mm).

We also studied the stability of the EMI SE values and the electrical conductivity (parallel direction) of GNR and MGNR composites under different cyclic stretching fatigue tests. The GNR and MGNR samples were cyclically stretched 250, 500, 1000, 1500, and 2000 times on a tensile fatigue testing machine (MTS810, MTS Corporation, Eden Prairie, MN, USA) at a strain of 25%. Some fractures in the MGNR-6 samples only occur after cyclic stretching more than 1000 times. This may be due to the addition of Fe_3_O_4_ particles, which decrease the mechanical properties of the composites. Therefore, the MGNR-6 composites were only cyclically stretched for 250 and 500 cycles. As shown in Figure 8a, MGNR composites have better EMI shielding stability performance compared with GNR composites (after 500 stretching cycles, the average EMI SE value of MGNR composites at all frequencies decreases by only 1.4%, while that of the GNR composites decreases by approximately 12.5%). However, the electrical conductivity (the *R*/*R*_0_ value) of MGNR composites exhibits more changes compared with GNR composites, which can be seen in Figure 8b. This is also due to the fact that cyclic stretching destroys the material’s segregated network structure and the Fe_3_O_4_ particles prevent the electronic transmission between the conductive rGO nano-platelets. This is the same result as the one we observed in the previous tensile permanent deformation experiment. In conclusion, the addition of Fe_3_O_4_ particles offers the composites better stability of EMI shielding performance but worse electrical conductivity robustness.

**Figure 9:**
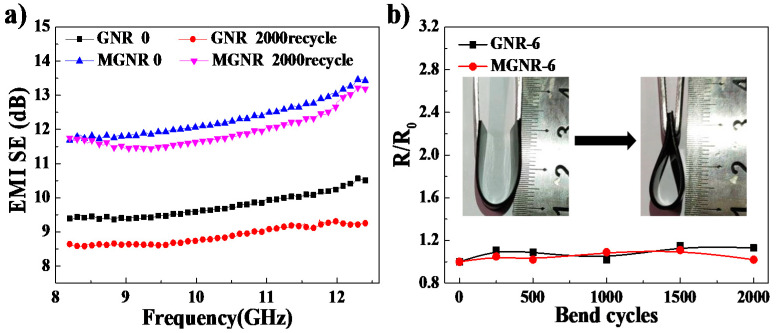
(**a**) The EMI SE of the MGNR-6 and GNR-6 composites before and after bending for 2000 cycles; and (**b**) normalized electrical resistance as a function of bend cycles for MGNR-6 and GNR-6 composites (the thickness was 0.6 mm).

Additionally, the reliability of EMI SE and electrical conductivity under cyclic bending are also important to the flexibility material and the MGNR-6 and GNR-6 samples underwent cyclic bending at a certain bending frequency (3 Hz) and angle (Figure 9b) for 250, 500, 1000, 1500, and 2000 cycles. The results of the stability of the EMI SE and electrical conductivity under cycle bending are shown in Figure 9. The results show that the MGNR-6 composites maintain bendability as well as GNR-6 composites do. After hundreds of bending cycles, the EMI shielding performance of both MGNR-6 and GNR-6 composites decreased to a certain extent (Figure 9a). However, compared with GNR composites (average decrease of approximately 9%), MGNR composites (average decrease of approximately 2.9%) exhibit better EMI shielding performance based on our results. Figure 9b displays the electrical resistance change (*R/R*_0_) as a function of bending cycles. Interestingly, the *R/R*_0_ of MGNR and GNR both remain very stable with no more than a 15% rise even after 2000 cycles. This indicates that bending experiments will not damage the segregated network of composites and MGNR composites can be used in flexible bending electronics.

### 3.3. Multiple Sensing Properties of the MGNR Composites

Reliable and excellent sensing properties are very important for flexible, wearable electronic devices. Through the research in the previous section, we found that the MGNR composites have a higher electrical resistance change under the same tensile permanent deformation conditions, which provides a good foundation for the preparation of composites with good sensing properties.

**Figure 10:**
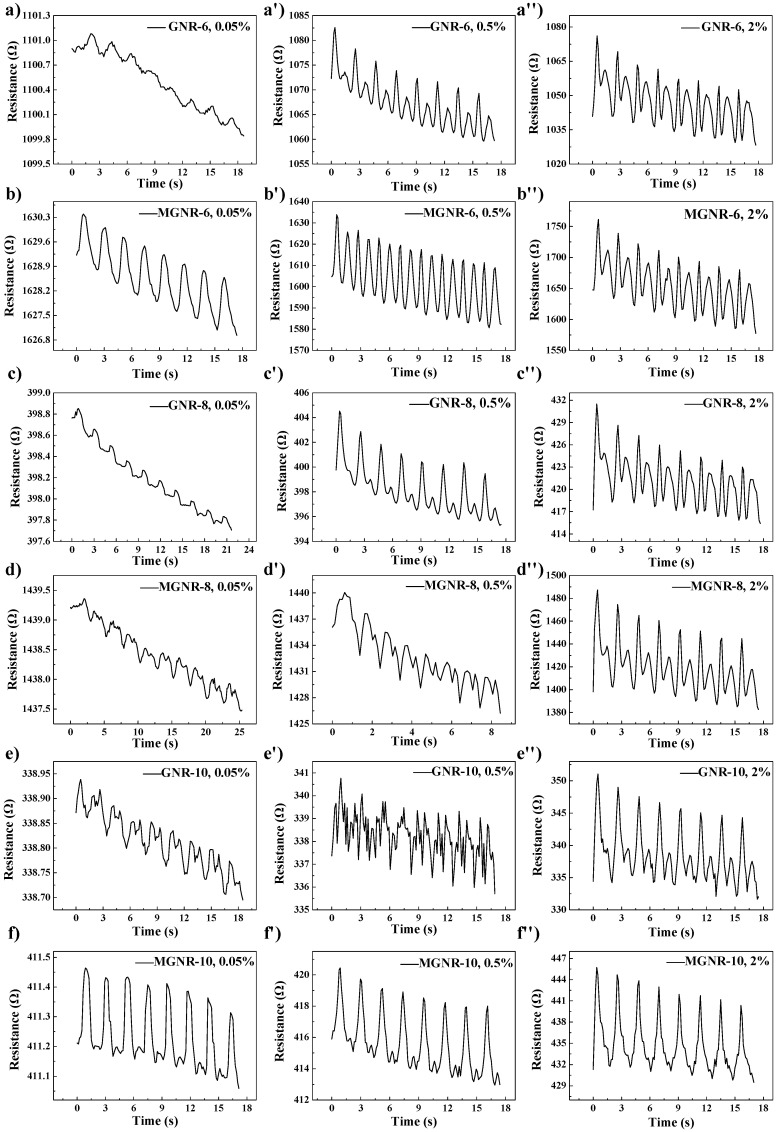
The change of resistance for the cyclic stretching in (**a**) GNR-6; (**b**) MGNR-6; (**c**) GNR-8; (**d**) MGNR-8; (**e**) GNR-10; and (**f**) MGNR-10 for different strain.

We first studied the sensing properties of different GNR and MGNR composites under different cyclic tensile strains (0.05%, 0.5%, and 2%) and the results are shown in Figure 10. Among them, the resistance changes in GNR composites are not obvious under low strain, while an obvious resistance change occurs under high strain. However, MGNR-6 and MGNR-10 composites have stable and obvious resistance changes under low strain (0.05%), indicating their excellent sensing performance. Except for MGNR-10 composites, the other GNR and MGNR composites all display small new peaks in the same cycle under high strain. This may be due to the segregated network structure in the composites, which cannot be recovered in time during the stretching and recovering process. The delayed recovery phenomenon of the segregated network leads to the appearance of such peaks [51,52,53]. From the above results, it can be seen that the MGNR-10 composites have the most stable and obvious resistance change, meaning they show the best sensing performance under cyclic tensile strain.

**Figure 11:**
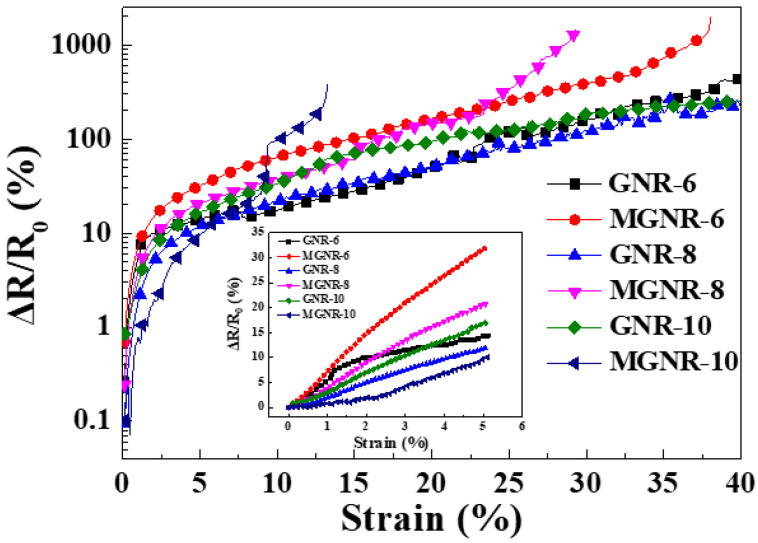
Relative resistance change (Δ*R/R*_0_) as a function of tensile strain in different composites.

The sensing performance of a material is usually characterized by its gauge factor (*GF*) [30,32]. Figure 11 shows the resistance changes in different GNR and MGNR composites under various tensile strains. From Equation (1), it can be seen that the GF of different materials is the slope value of the resistance change/strain curves in Figure 11. At low strain (<5%), the slope of the MGNR-6 composites is the greatest, indicating that the *GF* of MGNR-6 is the greatest in a small strain range. However, as the strain increases (5–10%), the *GF* of the MGNR-10 composites also increases. Within a certain strain range, the *GF* of the MGNR composites is larger than that of the GNR composites, indicating that the MGNR composites have better sensing performance compared with GNR composites (when the strain is 10%, the *GF* of the MGNR-10 composite is 14.13, while the *GF* of the GNR-10 composites is only 6.21). It is worth noting that within a certain range of elongation, the relative resistance change rate of MGNR composites will show a sudden increase. This is because the structure of the segregated network in MGNR composites will be damaged under the tensile strain, thus causing a significant change in the resistance. Additionally, with an increase in the Fe_3_O_4_ content, the elongation that produces this change is greatly reduced.

**Figure 12:**
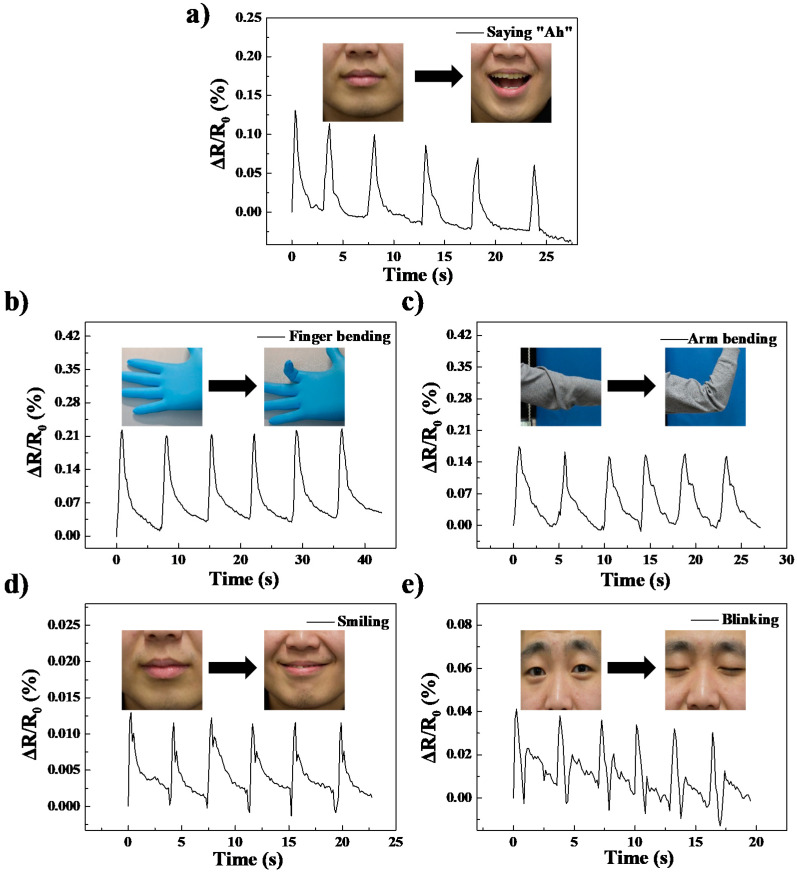
The recorded relative resistance change in the electronic sensor with the volunteer performing different motions: (**a**) saying ‘Ah’; (**b**) finger bending; (**c**) arm bending; (**d**) smiling; and (**e**) blinking.

The significant sensing properties of MGNR are suitable for skin-wearable sensors for real-time physiological and motion monitoring. Since the strain sensing properties of MGNR-10 composites are the best in terms of sensing stability and GF value among all the composites, we selected MGNR-10 composites for use in wearable applications to test the sensing properties of human motion such as finger bending, wrist bending, speaking, smiling, and blinking. Figure 12 shows the relative resistance change in the aforementioned motions and it can be seen that the MGNR-10 materials have stable and repeatable resistance signal changes in different human motions (from small facial muscle changes to large wrist bending or joint movements) because the segregated network in MGNR-10 composites can be more or less changed under these motions. From the above results, it can be seen that the MGNR composites have the potential to be used as a new flexible electronic material to monitor various behaviors of the human body, including human physiological signals and different body motions.

## 4. Conclusions

Magnetic MGNR composites with a segregated network were prepared by electrostatic self-assembly of Fe_3_O_4_ and graphene oxide followed by mixing with natural rubber latex and being in situ reduced by hydrazine hydrate. Fe_3_O_4_ nanoparticles were deposited on an rGO sheet layer and the segregated network provides the MGNR composites with excellent EMI shielding properties. Furthermore, the existence of Fe_3_O_4_ nanoparticles gives the MGNR composites outstanding EMI shielding stability under different tensile permanent deformation, cyclic stretching, and cyclic bending (the EMI SE value is reduced by no more than 2.9%). However, the EMI shielding performance of GNR composites has a certain degree of decline after the same treatment, by approximately 16% in the worst case. The Fe_3_O_4_ anchored on the segregated graphene network gives the composites a higher resistance change under different tensile strains, which can give the MGNR composites better sensing performance, even in the case of cyclic stretching with very low strain (0.05%). Resistance signal changes can also be stably and repeatedly monitored by the MGNR-10 composites when they are used to detect human motions such as finger bending, wrist bending, speaking, smiling, and blinking, indicating that the MGNR composites can be used in future flexible, wearable electronic devices.

## Data Availability

Not applicable.

## Supplementary Materials

The following are available online at https://www.mdpi.com/article/10.3390/polym13193277/s1, Figure S1. The effect of rGO content on the electrical conductivity of MGNR and GNR composites. Figure S2. (a) EMI SE of the MGNR and GNR composites with rGO content as a function of frequency. (b) Shielding by reflection, absorption, and total shielding of GNR nanocomposites. (c) Shielding by reflection, absorption, and total shielding of MGNR composites. (d) Effective absorb-ance of the MGNR and GNR composites. The thickness of the sample was 2 mm. Figure S3. Effect of the thickness on the EMI SE of MGNR-6 composites.
