# Supplementary material for: Constructing a Segregated Magnetic Graphene Network in Rubber Composites for Integrating Electromagnetic Interference Shielding Stability and Multi-Sensing Performance
Source: Polymers (Basel). 2021 Sep 26;13(19):3277. doi: 10.3390/polym13193277 (PMC8512318; doi:10.3390/polym13193277)
Supplement: Supplementary file 1 [file polymers-13-03277-s001.zip › polymers-1381089-supplementary.pdf]

Supporting Information

Constructing segregated magnetic graphene network in rubber composites for integrating electromagnetic interference shielding stability and multi-sensing performance

Jian Wang [1,*], Baohua Liu [1], Yu Cheng [2], Zhenwan Ma [2], Yanhu Zhan [3,*] and Hesheng Xia [2,*]

1. College of Food and Biological Engineering, Chengdu University, Chengdu 610106, China

2. State Key Laboratory of Polymer Materials Engineering, Polymer Research Institute, Sichuan University, Chengdu 610065, China

3. School of Materials Science and Engineering, Liaocheng University, Liaocheng 252059, China

Characterization

Electrical conductivity was detected by employing a picometer (Keithley 2400) system. The total EMI SE (SE_{Total}), absorption shielding (SE_A), reflection shielding (SE_R), multiple reflections shielding (SE_M) and effective absorbance (A_{eff}) can be obtained by using the above equations.

From the S_{11} and S_{21}, the power coefficients of reflectivity (R), transmissivity (T), and absorptivity (A) can be obtained using equation (S1)~(S3)[1,2]

$$R = |S_{11}|^2 \tag{S1}$$

$$T = |S_{21}|^2 \tag{S2}$$

$$\mathrm{A = 1 - R - T} \tag{S3}$$

Therefore, the effective absorbance (A_{eff}) can be described as [3]

$$A_{eff} = A/(A+T) \tag{S4}$$

The total EMI SE (SE_{Total}) was also calculated, and it was defined as the logarithmic ratio of incoming (P_{in}) to outgoing power (P_{out}) of electromagnetic radiation[4].

$$SE_{Total} = -10lg(\frac{P_{out}}{P_{in}}) = SE_R + SE_A + SE_M \tag{S5}$$

where SE_A, SE_R, and SE_M are the absorption shielding, reflection shielding, and multiple reflections shielding, respectively. The SE_M usually can be neglected when SE_{Total}>10 dB. Thus,

$$SE_{Total} = SE_R + SE_A \tag{S6}$$

where SE_R and SE_A can be obtained according to the following formula[3,4]:

$$SE_R = -10lg(1-R) \tag{S7}$$

$$SE_A = -10lg(\frac{T}{1-R}) \tag{S8}$$

Electrical conductivity of MGNR and GNR composites

The electrical conductivity of the MGNR and GNR composites will affect their EMI shielding properties and the electrical conductivity should be tested firstly. Figure S1 shows the effect of rGO content on the conductivity of MGNR and GNR composites. It is clear that the electrical conductivities of both GNR and MGNR composites increase with the addition of the rGO content. It is also worth noting that with the same amount of rGO, the conductivity of MGNR composite is lower than that of GNR composite. This is because the non-conductive Fe_3O_4 nanoparticles anchored on the surface of the rGO flakes will prevent the direct contact of the conductive rGO nano-platelets, resulting in a decrease in the conductivity of the MGNR composites. Although the differences between electrical conductivities of MGNR and GNR still exist, they are levelled down when rGO content increases. This indicating that the rGO segregated network play a main role in the electrical conductivity of GNR and MGNR composites and the negative effect of Fe_3O_4 on the electrical conductively at high rGO content will be weakened.

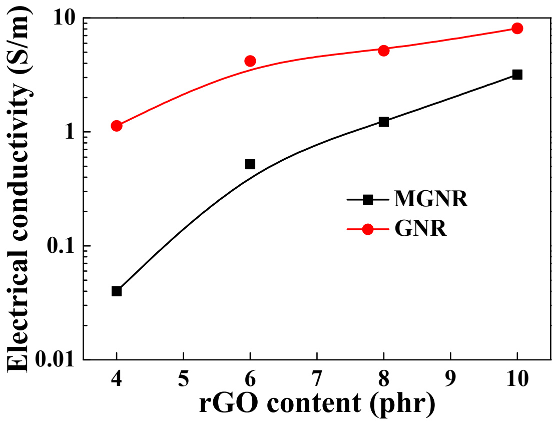

Figure S1. The effect of rGO content on the electrical conductivity of MGNR and GNR composites.

EMI shielding behaviors of MGNR and GNR composites

Figure S2a shows the EMI SE values of MGNR and GNR composites with the microwave

frequency range of 8.2–12.4 GHz (The thickness of all the samples were 2mm). The EMI SE values of all samples are frequency independent in the measured bands. It is clear that EMI SE values of both MGNR and GNR composites increase with the growth of rGO content. This is because the rGO with the segregated conductive network brings more high free charge carriers and high electric dipoles to the composites [1, 5]. It is also worth noting that under the same rGO content and same frequency, the EMI SE value of MGNR composites is much higher than that of GNR composites. Specifically, the EMI SE value of the MGNR-10 composites is 42.6 dB at 8.5 GHz, while the GNR-10 composites is only 32.4 dB at the same frequency. It is also surprising that the EMI SE value of MGNR-4 composites is higher than the EMI SE value of GNR-10 composites when the frequency is below 9GHz, and even the average EMI SE values are similar between MGNR-4 and GNR-10 composites under all the frequencies. This indicating that the addition of Fe_3O_4 can significantly improve the EMI shielding performance of the composites. This is because the Fe_3O_4 particles bring the composites more magnetic field interactions with natural resonance, exchange resonance, and eddy currents [6, 7]. Also, the addition of Fe_3O_4 can enhance the interface polarization relaxation between the fillers and rubber matrix, which will increase the transmission path of electromagnetic waves between composite materials, and the possibility of attenuation of incident waves increases consequently [8-10].

To understand the specific EMI shielding mechanism and performance of MGNR composites, the SE_R and SE_A value which presents the reflection and absorption EMI SE effect were calculated according to scattering parameters [1, 7, 11]. The SE_R and SE_A values of GNR and MGNR composites were shown in Figure S2b and Figure S2c respectively. It is noticed that the value of the SE_{Total} and SE_A enhanced with the increase of the rGO content from 4phr to 10 phr, also with the same increasing trend. On the other hand, the SE_R value didn't change so much when the rGO content increased. Form this result, it is the absorption efficiency, but not the reflection efficiency, that contributes more to the EMI SE of MGNR composites, absorbing most of the electromagnetic radiation, dissipated in the form of heat [12, 13]. As shown in Figure S2d, all of the contribution of SE_A to the total EMI SE of MGNR composites is more than 99.93% , much larger than that of GNR composites (99.36% $< A_{eff} <$ 99.9%). The addition of Fe_3O_4 promotes the controllable magnetic permeability properties of MGNR composites, with the

low reflection and high absorption of penetrated electromagnetic radiation by ohmic and magnetic losses [14, 15]. Above all, the MGNR composites effectively absorb and convert the electromagnetic energy into thermal or other energy. Therefore, they can have excellent EMI shielding performance [1, 14].

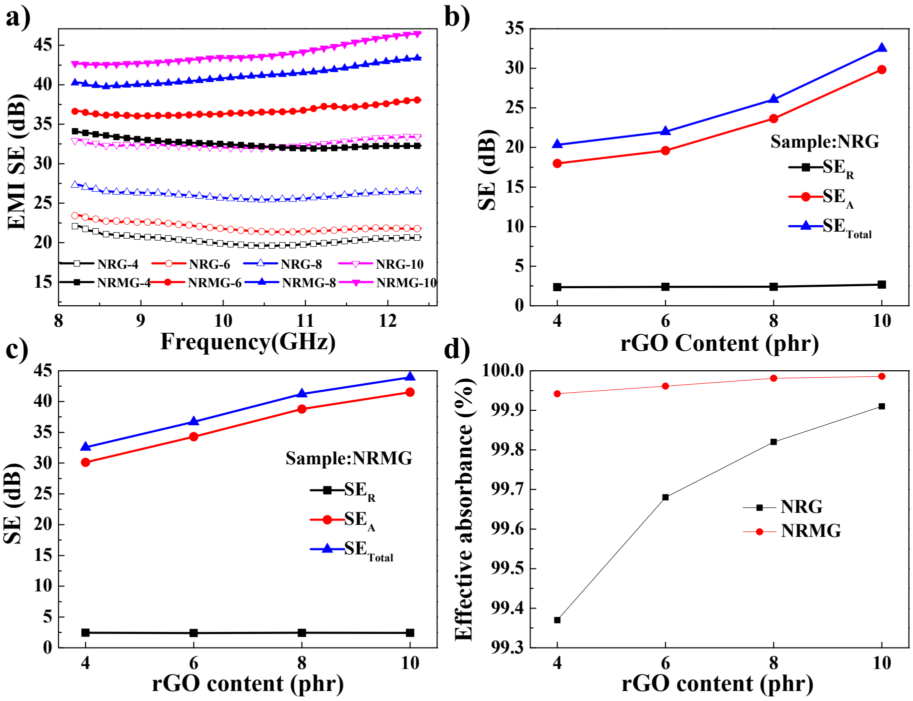

Figure S2. (**a**) EMI SE of the MGNR and GNR composites with rGO content as a function of frequency. (**b**) Shielding by reflection, absorption, and total shielding of GNR nanocomposites. (**c**)Shielding by reflection, absorption, and total shielding of MGNR composites. (**d**) Effective absorbance of the MGNR and GNR composites. The thickness of the sample was 2 mm.

The effect of sample thickness on the EMI shielding performance of the composites should also be considered because the thickness is also the main factor that will affect EMI SE value [15–17]. The EMI SE value of the MGNR-6 composites with different thickness from 0.6mm to 2 mm was shown in Figure S3. With the increase of the thickness, the EMI SE of MGNR-6 composites increases from 13.5 to 38.1dB at 12.4 GHz. This is because more Fe_3O_4 and rGO can effectively interact with the incident electromagnetic microwaves. So it is significant to better evaluate the EMI shielding performance of the composites by using the specific EMI SE (EMI SE divided by sample thickness) [1,18]. The specific EMI SE of MGNR-10 composites achieves 21.3 $dB\cdot mm^{-1}$, which can have the competitive EMI shielding performance with the reported properties of polymer/rGO or polymer/Fe_3O_4@rGO composites[7, 15, 19-21].

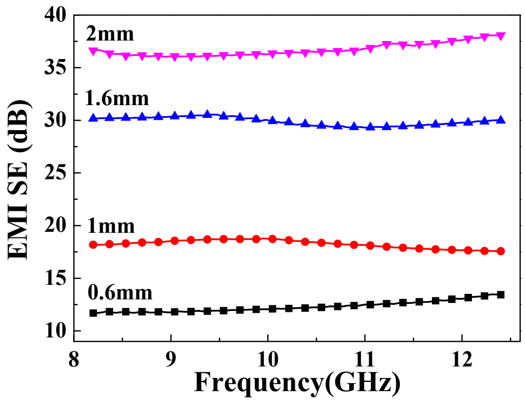

Figure S3. Effect of the thickness on the EMI SE of MGNR-6 composites.

References

1. Zhan, Y.H.; Wang, J.; Zhang, K.Y.; Li, Y.C.; Meng, Y.Y.; Yan, N.; Wei, W.K.; Peng, F.B.; Xia, H.S. Fabrication of a Flexible Electromagnetic Interference Shielding Fe_3O_4@Reduced Graphene Oxide/Natural Rubber Composite with Segregated Network. *Chem. Eng. J.* **2018,** 344, 184–193.
2. Kwon, S.; Ma, R.; Kim, U.; Choi, H.R.; Baik, S. Flexible electromagnetic interference shields made of silver flakes, carbon nanotubes and nitrile butadiene rubber. *Carbon* **2014,** 68, 118-124.
3. Hong, Y.; Lee, C.; Jeong, C.; Lee, D.; Kim, K.; Joo, J. Method and apparatus to measure electromagnetic interference shielding efficiency and its shielding characteristics in broadband frequency ranges. *Rev. Sci. Instrum.* **2003,** 74, 1098-1102.
4. Zhang, K.; Yu, H.O.; Shi, Y.D.; Chen, Y.F.; Zeng, J.B.; Guo, J.; Wang, B.; Guo, Z.H,; Wang, M. Morphological regulation improved electrical conductivity and electromagnetic interference shielding in poly (L-lactide)/poly (ε-caprolactone)/carbon nanotube nanocomposites via constructing stereocomplex crystallites. *J. Mater. Chem. C.* **2017,** 5, 2807-2817.
5. Pawar, S.P.; Marathe, D.A.; Pattabhi, K.; Bose, S. Electromagnetic interference shielding through MWNT grafted Fe3O4 nanoparticles in PC/SAN blends. *J. Mater. Chem. A.* **2015,** 3, 656-669.
6. Wang, L.; Wu, Y.; Wang, Y.; Li, H.F.; Jiang, N.S.; Niu, K.M. Laterally compressed graphene foam/acrylonitrile butadiene styrene composites for electromagnetic interference shielding. *Compos. Part. A-Appl. S.* **2020,** 133, 105887.
7. Sharif, F.; Arjmand, M.; Moud, A.A.; Sundararaj, U.; Roberts, E.P.L. Segregated Hybrid Poly(methyl methacrylate)/Graphene/Magnetite Nanocomposites for Electromagnetic Interference Shielding. *ACS. Appl. Mater. Inter.* **2017,** 9, 14171-14179.
8. Guo, Z.Z.; Ren, P.G.; Dai, Z.; Zong, Z.; Zhang, F.D.; Jin, Y.L.; Ren, F. Construction of interconnected and oriented graphene nanosheets networks in cellulose aerogel film for high-efficiency electromagnetic interference shielding. *Cellulose* **2021,** 28, 3135-3148.
9. Xiao, S.S.; Mei, H.; Han, D.Y.; Dassios, K.G.; Cheng, L.F. Ultralight lamellar amorphous carbon foam nanostructured by SiC nanowires for tunable electromagnetic wave absorption. *Carbon* **2017,** 122, 718-725.
10. Mei, H.; Han, D.; Xiao, S.S.; Ji, T.M.; Tang, J.; Cheng, L.F. Improvement of the electromagnetic shielding properties of C/SiC composites by electrophoretic deposition of carbon nanotube on carbon fibers. *Carbon* **2016,** 109, 149-153.

11. Jia, L.C.; Yan, D.X.; Cui, C.H.; Ji, X.; Li, Z.M. A Unique Double Percolated Polymer Composite for Highly Efficient Electromagnetic Interference Shielding. *Macromol. Mater. Eng.* **2016,** 301, 1232-1241.
12. Mondal, S.; Ganguly, S.; Rahaman, M.; Aldalbahi, A.; Chaki, T.K.; Khastgir, D.; Das, N.C. A strategy to achieve enhanced electromagnetic interference shielding at low concentration with a new generation of conductive carbon black in a chlorinated polyethylene elastomeric matrix. *Phys. Chem. Chem. Phys.* **2016,** 18, 24591-24599.
13. Wei, L.; Ma, J.; Zhang, W.; Bai, S.L.; Ren, Y. Zhang, L. Wu, Y.K.; Qin, J.B. pH triggered hydrogen bonding for preparing mechanically strong, electromagnetic interference shielding and thermally conductive waterborne polymer/graphene@polydopamine composites. *Carbon* **2021,** 181, 212-224.
14. Chen, C.Y.; Pu, N.W.; Liu, Y.M.; Huang, S.Y.; Wu, C.H.; Ger, M.D.; Gong, Y.J.; Chou, Y.C. Remarkable microwave absorption performance of graphene at a very low loading ratio. *Compos. Part B-Eng*. **2017,** 114, 395-403.
15. Chen, Y.; Li, J.Z.; Li, T.; Zhang, L.K.; Meng, F.B. Recent advances in graphene-based films for electromagnetic interference shielding: Review and future prospects. *Carbon* **2021,** 180, 163-184.
16. Chen, Y.; Zhang, H.B.; Yang, Y.; Wang, M.; Cao, A.; Yu, Z.Z. High-Performance Epoxy Nanocomposites Reinforced with Three-Dimensional Carbon Nanotube Sponge for Electromagnetic Interference Shielding. *Adv. Funct. Mater.* **2016,** 26, 447-455.
17. Lee, S.H.; Kang, D.; Oh, I.K. Multilayered graphene-carbon nanotube-iron oxide three-dimensional heterostructure for flexible electromagnetic interference shielding film. *Carbon* **2017,** 111, 248-257.
18 Shen, B.; Li, Y.; Zhai, W.T.; Zheng, W.G. Compressible Graphene-Coated Polymer Foams with Ultralow Density for Adjustable Electromagnetic Interference (EMI) Shielding. *ACS. Appl. Mater. Inter.* **2016,** 8, 8050-8057.
19. Kashi, S.; Gupta, R.K.; Baum, T.; Kao, N.; Bhattacharya, S.N. Morphology, electromagnetic properties and electromagnetic interference shielding performance of poly lactide/graphene nanoplatelet nanocomposites. *Mater. Design.* **2016,** 95, 119-126.
20. Shen, B.; Zhai, W.T.; Tao, M.M.; Ling, J.Q.; Zheng, W.G. Lightweight, Multifunctional Polyetherimide/Graphene@Fe_3O_4 Composite Foams for Shielding of Electromagnetic Pollution. *ACS. Appl. Mater. Inter.* **2013,** 5, 11383-11391.
21. Wan, Y.J.; Zhu, P.L.; Yu, S.H.; Sun, R.; Wong, C.P.; Liao, W.H. Ultralight, super-elastic and volume-preserving cellulose fiber/graphene aerogel for high-performance electromagnetic interference shielding. *Carbon* **2017,** 115, 629-639.
